# Biopsychosocial inequality, active lifestyle and chronic health conditions: a cross-sectional National Health Survey 2013 in Brazil

**DOI:** 10.1038/s41598-021-03549-5

**Published:** 2021-12-14

**Authors:** Marcello Barbosa Otoni Gonçalves Guedes, Rodolpho Nunes Araújo, Lídia Reniê Fernandes da Silva, Diego Neves Araujo, Sanderson José Costa de Assis, Thais Sousa Rodrigues Guedes, Eldys Myler Santos Marinho, Clécio Gabriel de Souza, Johnnatas Mikael Lopes

**Affiliations:** 1 Universidade Federal do Rio Grande do Norte, Desembargador José Gomes da Costa, 1884, Capim Macio. Natal, Rio Grande do Norte, 59082140 Brazil; 2 Universidade Federal do Vale do São Francisco, Paulo Afonso, Bahia Brazil; 3 Unifacisa Centro Universitário, Campina Grande, Paraíba Brazil; 4 Uninassau Centro Universitário, Rio Grande do Norte, Brazil

**Keywords:** Diseases, Health care, Rheumatology

## Abstract

This study estimated the biopsychosocial factors related to active physical behavior in the Brazilian population with and without chronic non-transmissible disease (NCD). Cross-sectional study of the National Health Survey (NHS) in Brazil, with 60,202 individuals in 2013. Participants were randomly selected by complex sampling. The outcome was physically active behavior measured by performing a minimum of 150 min of physical exercise per week. The independent variables were social and psychological characteristics, lifestyle and health. Cox regression was applied to estimate the prevalence ratio (PR). There are 29,666 (48.3%; 95% CI 47.0–50.0) participants reported having NCD. Not being a smoker or alcoholic, living in an urban area (PR = 1.44; CI95% 1.23–1.68/PR = 1.38; CI95% 1.08–1.75), having informal social support (PR = 1.26; CI95% 1.10–1.44/PR = 1.19; CI95% 1.05–1.34), A social class (PR = 0.43; CI95% 0.25–0.73/PR = 0.46; CI95% 0.26–0.80), high schooling (PR = 0.34; CI95% 0.23–0.51/PR = 0.33; CI95% 0.24–0.46) as well as paid work (PR = 0.87; CI95% 0.78–0.96/PR = 0.89; CI95% 0.79–0.99) are more associated with active lifestyle in both groups. However, only in the group without NCD, the male sex (PR = 1.42; CI95% 1.28–1.57), no having some disability (PR = 1.31; CI95% 1.03–1.66) and having private health insurance (PR = 1.26; CI95% 1.13–1.41) were more associated with active behavior, while in the group with NCD, being elderly (PR = 1.22; CI95% 1.05–1.42), not be white (PR = 0.85; CI95% 0.77–0.95) and not having restful sleep (PR = 1.23; CI95% 1.08–1.40) are associated with active lifestyle. People with and without NCD in Brazil have very close active behavior, however, some biopsychosocial factors such as: sex, age, lifestyle, socioeconomic level are unevenly associated with the active lifestyle in the groups. Thus, therapeutic or preventive proposals as well as public policies for health promotion must observe these distinctions when elaborating their actions.

## Introduction

The World Health Organization (WHO) recommends performing at least 150 min of moderate physical activity or 75 min of vigorous physical activity weekly for an individual to be considered physically active and to obtain health benefits. Furthermore, adding resistance, flexibility and balance training are essential to enhance the benefits generated by physical exercise, including the prevention and decreased progression of chronic non-communicable diseases (NCDs) and deaths for all causes^1^.

People with NCDs have slow progression of their conditions and they are generally composed solely or a combination of by mental, cardiovascular, metabolic, respiratory, neoplastic and musculoskeletal diseases with multimorbidity being a more worrying condition^2^. In 2016, they were responsible for 71% of deaths worldwide, the highest risk of death in middle and low-income countries^3^. In this country, 74% of deaths are due to NCDs and about 45% of the adult population report having at least one of these conditions^4^.

Global estimates indicate physical inactivity as accountable for 7.2% of death causes by NCDs and it generates a burden of 69% of all deaths from NCDs coming from middle-income countries^5^. On an individual basis, it is considered that sedentary people have a 20–30% higher risk of death from NCDs when compared to physically active people. Regular physical activity has a considerable role in preventing and reducing the progression of NCDs, therefore it is included within the Strategic Action Plan for Tackling Chronic Non-communicable Diseases in Brazil 2011–2022^6–9^.

Despite so many benefits, the number of people who are considered physically active in Brazil is low. About 45% of the Brazilian population is considered sedentary, because of multiple factors, such as the lack of social and/or behavioral support^10,11^. However, people with NCDs greatly benefit from regular active behavior, that helps to mitigate the disabilities produced by morbidity and, sometimes, reversing their progression.

However, access to the practice of regular physical activity is not equal in society, it depends on individual^12^ and contextual^13^ characteristics, especially in developing countries. In addition to the several factors that may be associated with inequitable access to physical activity, adherence to physical activity by Brazilians with NCDs is mediated by factors such as income and education^14^.

Social determinants address, in general, the living and working conditions of individuals that somehow determine their health^15^ and need to be analyzed and incorporated in public policies for collective and individual management of NCDs and physical activity practice^16^. Strictly, when it comes to health, identifying these determinants and discussing disparities in living and working conditions, access to health services and availability of public health resources are essential to reduce health inequities and to improve the living conditions of the population^17^.

There is strong evidence that high socioeconomic levels are associated with a higher level of leisure-time physical activity and, therefore, it is associated with better health in general, whereas most physical activity is work-related among the population of lower socioeconomic levels^18,19^. Social determinants strongly influence healthy behavior, maintaining or deepening social disparities regarding self-care in health, responsible for NCD control and their morbidity and mortality rates. Studies show that inequalities in education and race are related to the presence of chronic diseases^19–21^ and may modulate active behavior together with other factors^19^. Therefore, understanding the conditioning factors of active behavior in people without NCDs is as important as in those with NCDs in order to outline specific actions for primary prevention in this population and manage previously installed conditions.

Hence, it is necessary to estimate the relationship that biopsychosocial factors establish in the physically active behavior of people with and without NCDs. We hypothesize that biopsychosocial conditions have different relationships between people with and without NCDs, which may possibly create a great vicious cycle of maintaining sedentary behavior in these subpopulations.

## Methods

This is a cross-sectional study from the Brazilian National Health Survey (NHS) database, developed in 2013 by the Brazilian Institute of Geography and Statistics (IBGE) in partnership with the Oswaldo Cruz Foundation. This research was approved by the National Committee for Ethics in Research under the number 10853812.7.0000.0008. Data from the National Health Survey in Brazil is publicly available and can be accessed at: https://www.ibge.gov.br/estatisticas/sociais/saude/9160-pesquisa-nacional-de-saude.html?=&t=o-que-e. The authors had no access to any identifying information of participants-only aggregated data was analysed.

The preparation of this manuscript followed the criteria guided by the STrengthening the Reporting of OBservational studies in Epidemiology (STROBE) guidelines.

NHS population includes permanent residents, grouped in census sections in state capitals, metropolitan regions and cities, and it excludes indigenous villages, military bases and barracks, camps, inns, boats, penitentiaries, penal colonies, prisons, jails, orphanages, convents and hospitals.

The sample size was determined from the 2010 Demographic Census conducted by IBGE, the population groups of interest were selected and a sample size of 81,357 individuals aged over 18 years was estimated. Sampling took place by conglomerates, first stage was the census sector, followed by households in the sector and then the resident by simple random sampling. The survey was conducted by trained interviewers that visited each selected household to obtain participant's information. For more information about the PNS 2013 sampling plan, see Szwarcwald et al.

Participants were divided into two groups (non-NCD and NCD) and responded to a research instrument organized by thematic modules from A to W. The modules that involve individual characteristics of residents, such as demographic, social, lifestyle, health perception, chronic illnesses and household characteristics were analyzed. The independent variables were grouped according to the NHS modules and were listed in Table 1.

**Table 1:** Characterization of the sample according to the health condition for chronic non-communicable diseases (NCDs).

Variables		Total	% (CI-95%)	Non-NCD physical activity practitioner					Total	% (CI-95%)	NCD physical activity practitioner				
				n	%	CI-95%	x^2^	p			n	%	CI-95%	x^2^	p
Sex	Male	14,997	51.6 ( 50.6–52.6)	3484	23.5	(22.3–24.8)	**44.47**	< 0.001	10,923	38.6 (37.6–39.6)	1995	17.4	(16.2–18.7)	**0.13**	0.714
	Female	15,539	48.4 (47.4–49.4)	2654	17.7	(16.5–18.9)			18,743	61.4 (60.4–62.4)	3079	17.5	(16.5–18.6)		
Elderly	No	27,941	91.5 (90.9–92.1)	5829	21.5	(20.6–22.5)	**31.92**	< 0.001	21,084	71.0 (69.9–71.9)	3919	19.1	(18.1–20.1)	**41.92**	**< 0.001**
	Yes	2595	8.5 (7.9–9.1)	309	11.9	(9.7–14.4)			8582	13.5 (28.1–30.1)	1155	13.5	(12.13–14.8)		
Race	White	11,543	45.4(44.4–46.5)	2670	22.3	(21.0–23.6)	**15.52**	< 0.001	12,563		2378	18.8	(17.6–20.0)	**9.92**	**0.002**
	Non-white	18,993	54.6 (53.5–55.6)	3468	19.4	(18.3–20.6)			17,103		2696	16.2	(15.1–17.3)		
Education	Illiterate	1635	7.4 (5.2–10.6)	97	7.4	(5.2–10.6)	**82.10**	< 0.001	2809	8.2 (7.7–8.8)	161	6.2	(4.8–7.9)	**96.31**	**< 0.001**
	Literate	2153	6.8 (5.1–9.1)	140	6.8	(5.1–9.1)			5626	20.6 (19.7–21.6)	540	10.4	(9.1–11.8)		
	Elementary education	8384	13.1(11.6–14.8)	994	13.1	(11.6–14.8)			7771	25.8 (24.8–26.8)	953	12.7	(11.4–14.1)		
	High education	11,867	40.1 (39.1–41.2)	2677	23.5	(22.1–24.9)			8159	27.7 (26.8–28.6)	1744	21.3	(19.8–22.9)		
	Higher education	6497	20.8 (19.8–21.8)	2230	33.1	(30.1–35.6)			5301	17.7 (16.7–18.8)	1676	32.1	(29.8–35.2)		
Living with spouse	Yes	17,541	58.4 (57.4–59.5)	3023	17.2	(16.2–18.2)	**71.01**	< 0.001	16,981	64.6 (63.6–65.5)	2813	16.7	(15.7–17.6)	**6.395**	**0.011**
	No	12,995	41.6 (40.5–42.6)	3115	25.7	(24.2–27.3)			12,685	35.4 (34.5–36.4)	2261	19.0	(17.6–20.4)		
Paid work	Yes	19,647	65.0 (63.9–66.0)	4315	22.0	(21.0–23.0)	**19.90**	< 0.001	14,343	49.3 (48.3–50.3)	2791	19.4	(18.3–20.7)	**22.98**	**< 0.001**
	No	10,889	35.0 (34–36.1)	1813	18.3	(16.9–19.8)			15,323	50.7 (49.7–51.7)	2283	15.6	(14.5–16.7)		
Health Insurance	Yes	7708	28.0 (26.9–29.1)	2462	30.0	(28.2–31.8)	**180.81**	< 0.001	8660	32.9 (31.7–34.2)	2233	24.8	(23.3–26.3)	**155.30**	**< 0.001**
	No	22,828	72.0 (70.9–73.1)	3676	17.1	(16.1–18.1)			21,006	67.1 (65.8–68.3)	2841	13.9	(13.0–14.8)		
**BMI**							1.68	0.194						0.03	0.862
Drinking	No	17,629	55.9 (54.8–57.0)	2915	17.3	(16.3–18.4)	**87.12**	< 0.001	19,571	64.7 (63.6–65.7)	2824	14.7	(13.8–15.6)	**92.11**	**< 0.001**
	Yes	12,907	44.1 (43.0–45.2)	3223	25.0	(23.6–26.4)			10,095	35.3 (34.3–36.4)	2250	22.6	(21.1–24.1)		
Smoking	Yes	4332	14.2 (13.5–14.9)	565	13.9	(12.1–16.0)	**39.30**	< 0.001	4397	15.2 (14.5–15.9)	526	12.1	(10.6–13.9)	**34.36**	**< 0.001**
	No	26,204	85.8 (85.1–86.5)	5573	21.8	(20.9–22.8)			25,269	84.8 (84.1–85.5)	4548	18.4	(17.6–19.3)		
Informal social support	3–5 people	10,482	34.2 (33.2–35.3)	2212	21.7	(20.2–23.3)	**17.26**	< 0.001	9352	31.9 (30.9–32.8)	1676	18.6	(17.3–19.9)	**20.38**	**< 0.001**
	6–10 people	5533	19.2 (18.4–20.1)	1408	25.8	(23.7–28.0)			5677	20.6 (19.7–21.5)	1249	22.6	(19.7–21.5)		
	11–15 people	1464	5.2 (4.8–5.7)	395	23.0	(19.8–26.6)			1610	5.6 (19.9–26.9)	352	23.2	(19.9–26.9)		
	16–20 people	473	2.0 (1.7–2.4)	353	27.1	(21.1–34.0)			629	2.2 (1.9–2.5)	132	21.7	(16.6–27.9)		
	21 or more people	554	2.2 (1.8–2.6)	171	28.2	(21.9–35.4)			720	2.8 (2.5–3.2)	140	18.7	(13.8–24.9)		
	2 or less people	12,030	37.1 (36.0–38.2)	1832	16.0	(14.8–17.2)			11,678	36.9 (35.9–38.0)	1525	12.8	(11.7–13.9)		
Social Class	D/E	13,291	37.0 (35.9–38.1)	1675	14.1	(12.9–15.3)	**89.86**	< 0.001	13,452	37.3 (36.3–38.4)	1339	10.4	(9.5–11.4)	**111.91**	**< 0.001**
	C	13,036	46.9 (45.8–48.0)	2893	21.3	(20.0–22.6)			12,065	45.1 (44.0–46.2)	2322	17.4	(16.2–18.7)		
	B	4127	15.6 (14.7–16.6)	1522	33.5	(31.1–35.9)			4062	17.0 (15.9–18.2)	1373	32.5	(30.0–35.1)		
	A	82	0.5 (0.3–0.7)	48	63.2	(40.4–81.3)			87	0.6 (0.4–0.8)	40	39.3	(24.3–56.6)		
Depressive symptoms perception	No	23,325	78.4 (77.5–79.3)	4753	20.8	(19.8–21.8)	0.33	0.560	16,529	57.2 (56.1–58.3)	3011	19.1	(18.0–20.2)	**23.34**	**< 0.001**
	Yes	7211	21.6 (20.7–22.5)	1385	20.3	(18.6–22.1)	1		13,137	42.8 (41.7–43.9)	2063	15.4	(14.3–16.5)	1	
Behavioral lability perception	No	25,544	84.8 (84.1–85.6)	5226	21.1	(20.2–22.1)	**7.00**	0.008	19,747	67.4 (66.4–68.4)	3598	19.0	(18.0–20.0)	**34.50**	**< 0.001**
	Yes	4992	15.2 (14.4–15.9)	912	18.4	(16.5–20.5)			9919	32.6 (31.6–33.6)	1476	14.4	(13.2–15.6)	1	
Restful sleep	No	24,665	81.7 (80.8–82.6)	1337	19.3	(17.9–21.3)	2.48	0.115	17,685	60.4 (59.3–61.4)	1888	14.7	(13.6–16.0)	**30.20**	**< 0.001**
	Yes	5871	18.3 (17.4–19.2)	4801	21.0	(20.0–22.0)			11,981	39.6 (38.6–40.7)	3186	19.3	(18.2–20.4)	1	
General health perception	Good	24,638	82.2 (81.4–83.0)	5460	22.5	(21.5–23.5)	**52.99**	< 0.001	14,503	49.7 (48.7–50.8)	3317	23.0	(21.7–24.3)	**100.64**	**< 0.001**
	Regular	5328	16.2 (15.5–17.0)	647	13.0	(11.4–14.7)			11,869	40.1 (39.1–41.1)	1518	13.4	(12.2–14.5)		
	Poor	570	1.6 (1.3–1.8)	31	6.2	(3.7–10.1)			3294	10.1 (9.6–10.7)	239	6.8	(5.5–8.4)	1	
Disabilities	No	29,079	21.1 (94.8–95.7)	5927	21.1	(20.2–22.0)	**14.32**	< 0.001	25,599	18.4 (17.5–19.3)	4605	18.4	(17.5–19.3)	**30.77**	**< 0.001**
	Yes	1457	4.7 (4.3–5.2)	211	13.3	(10.5–16.8)			4067	12.0 (10.4–13.8)	469	12.0	(10.4–13.8)	1	
**Housing**															
	Urban	24,842	85.8 (85.1–86.5)	5538	22.3	(21.4–23.3)	130.89	< 0.001	24,403	87.1 (86.4–87.7)	4584	18.7	(17.8–19.6)	54.93	< 0.001
	Rural	5694	14.2 (13.5–14.9)	600	10.7	(9.5–12.2)			5263	12.9 (12.3–13.6)	490	9.4	(7.8–11.3)		

Significant values are in bold.

NCDs, Noncommunicable diseases; N, Number of cases in each category of the independent variable; %, percentage of people in each category of the independent variable; CI-95%, 95% confidence interval; PR, Prevalence ratio; BMI, Body Mass Index.

The outcome of the study was a minimal of 150 min of exercise per week, measured through self-report in the questioning: "In general, on the day you practice exercise or sports, how long does this activity last?". The individuals, based on these data, were dichotomized into active or sedentary. The independent variables were grouped according to the NHS modules. Demographic variables: sex (male or female); elderly (yes/no) who were considered those aged over 60 years; living with spouse (yes/no), race (white or non-white), paid work (yes/no) and social class (A, B, C and DE) where A is higher social class and DE is lower or deprived social class. The social class was generated from the Brazilian Economic Classification Criteria developed by the Brazilian Research Companies Association (ABEP)^17^.

The health variables were: number of NCDs only in NCD group; self-reported presence of sleep complaints based on the question "In the past two weeks, how often did you have problems for not feeling rested and willing during the day, feeling tired, without energy?", which also generated an ordinal variable with the responses: "no day", "less than half the days" and "more than half the days/almost every day" that were transformed into a dichotomous variable (yes/no) for restful sleep; disability for activities of daily living (yes/no). Disability measured by the presence of physical, mental and/or sensory disability (yes/no); Body Mass Index (BMI) was measured in kg/m^2^.

The health perception variables were general health perception (good, regular or poor), depressive symptoms perception (yes/no), and behavioral lability perception (yes/no). Lifestyle variables were smoking (yes/no) and drinking (yes/no). Social network variables were based on the question "how many family members or relatives and friends you feel at ease and can talk about almost everything?". This variable was stratified into less than 2 people, 3–5 people, 6–10 people, 11–15 people, 16–20 people and 21 or more people. Household variables were location of the household (urban or rural).

Data analysis took into account the complex sampling planning, where punctual and non-verbal estimates are influenced by the weighting of the sampling units of the last stage.

Data description was performed using prevalences and their respective 95% confidence intervals (95% CI). The inferential analysis for the association between the independent variables and the outcome was initially developed in the bivariate form or crude analysis of the prevalence ratio (PR) and its 95% CI. Subsequently, an adjusted model was created with the input of independent variables that revealed a probability less than 20% of the null hypothesis of the association to be true. The order of entry into the model was given by the hierarchical method, where proximal variables were included first and the most distal ones afterwards, according to a biopsychosocial approach. The Cox regression method was used, with adjustment for complex design for crude and adjusted analysis and Wald × 2 significance test for selection of independent variables related to the outcome. The crude and adjusted models were stratified for individuals with and without NCD, adopting an α ≤ 0.05.

## Results

In this study, 60,202 individuals were evaluated, 30,536 (51.7%; CI95% 51.0–52.5) of these reported not having any NCDs, whereas 29.666 (48.3%; CI95% 47.0–50.0) reported having at least one of them. We identified only 20.7% (CI95% 19.8–21.6) active individuals without NCDs and 17.5% (CI95% 16.7–18.3) with NCDs. More characteristics of these groups are shown in Table 1.

The groups differ proportionally regarding sex, with more women (61.4%) in the NCD group and more men (51.6%) in the non-NCD group. The NCD group is also composed of older people (13.5%) as well as people who report more depressive symptoms (42.8%), emotional lability (32.6%), poor health perception (10.1%) and disabilities (12.0%). People without NCD showed less restful sleep (18.3%). The educational level of the groups differs in terms of literate, elementary, high and higher education, where there is less education among individuals with NCD. In the non-NCD group, there are more people in paid work (65.0%) while in the NCD group there are more people with health insurance (32.9%). The groups are similar in terms of smoking and drinking alcohol, as well as in the composition of social classes, informal social support and housing (Table 1). The associations between independent variables and active lifestyle for individuals with and without NCDs were analyzed and we identified that all of them were related to at least one of the groups or both in the crude model (p ≤ 0.05), with the exception of BMI (Table 1).

In the adjusted model (Table 2), it is evident that household location, education level, having paid work, living alone, smoking, drinking and health perception are associated with an active lifestyle in both groups and are not factors that generate inequalities between people with and without NCDs, but inequalities in general population regarding the adoption of an active lifestyle.

**Table 2:** Adjusted model for the association between individual and contextual characteristics with active lifestyle in subjects with and without chronic non-communicable diseases (NCDs) in Brazil.

Parameter	Non-NCD				NCD			
	PR_crude_ (CI95%)	PR_Adj_	CI95%		PR_crude_ (CI95%)	PR_Adj_	CI95%	
**Sex**								
Male	**1.36 (1.24–1.50)**	**1.42**	**1.28**	**1.57**	0.98 (0.88–1.09)	0.94	0.83	1.06
Female	1	1				1		
**Elderly**								
No	**1.87 (1.50–2.32)**	1.23	0.98	1.55	**1.45 (1.29–1.62)**	**1.22**	**1.05**	**1.42**
Yes	1							
**Race**								
White	**1.21 (1.10–1.33)**	0.91	0.82	1.00	**1.17 (1.06–1.30)**	**0.85**	**0.77**	**0.95**
Non-white	1	1				1		
**Education**								
Illiterate	**0.94 (0.13–0.28)**	**0.34**	**0.23**	**0.51**	**0.16 (0.12–0.21)**	**0.33**	**0.24**	**0.46**
Literate	**0.17 (0.12–0.24)**	**0.28**	**0.20**	**0.40**	**0.28 (0.24–0.33)**	**0.52**	**0.42**	**0.63**
Elementary education	**0.34 (0.29–0.40)**	**0.54**	**0.45**	**0.65**	**0.35 (0.30–0.41)**	**0.62**	**0.52**	**0.75**
High education	**0.64 (0.57–0.71)**	**0.83**	**0.74**	**0.94**	**0.61 (0.54–0.69)**	**0.84**	**0.73**	**0.96**
Higher education	1	1			1	1		
**Living with spouse**								
Yes	**0.65 (0.59–0.72)**	**0.67**	**0.61**	**0.74**	**0.88 (0.79–0.97)**	**0.85**	**0.76**	**0.94**
No	1	1			1	1		
**Paid work**								
Yes	**1.26 (1.14–1.39)**	**0.87**	**0.78**	**0.96**	**1.28 (1.15–1.42)**	**0.89**	**0.79**	**0.99**
No		1				1		
**Health insurance**								
Yes	**1.95 (1.77–2.15)**	**1.26**	**1.13**	**1.41**	**1.89 (1.71–2.09)**	1.04	0.92	1.17
No	1	1			1	1		
BMI	0.99 (0.98–1.00)	0.99	0.98	1.00	1.00 (0.99–1.00)	1.00	0.99	1.00
**Drinking**								
Yes	**0.65 (0.59–0.71)**	**0.77**	**0.70**	**0.85**	**0.62 (0.56–0.68)**	**0.77**	**0.69**	**0.85**
No	1	1			1	1		
**Smoking**								
Yes	**0.60 (0.52–0.71)**	**0.64**	**0.553**	**0.76**	**0.63 (0.54–0.74)**	**0.69**	**0.58**	**0.81**
No	1	1			1	1		
**Informal social support**								
21 people or more	**1.42 (1.28–1.60)**	1.62	1.24	2.12	**1.49 (1.33–1.68)**	**1.19**	**1.05**	**1.34**
16–20 people	**1.72 (1.51–1.97)**	1.20	0.86	1.66	**1.79 (1.56–2.04)**	**1.37**	**1.19**	**1.58**
11–15 people	**1.53 (1.26–1.86)**	1.17	0.97	1.41	**1.88 (1.55–2.28)**	**1.53**	**1.27**	**1.85**
6–10 people	**1.82 (1.36–2.43)**	**1.26**	**1.10**	**1.44**	**1.79 (1.32–2.41)**	**1.46**	**1.07**	**1.99**
3–5 people	**1.93 (1.42–2.61)**	**1.17**	**1.049**	**1.31**	**1.51 (1.07–2.13)**	1.30	0.90	1.86
2 people or less	1	1			1	1		
**Social class**								
DE	**0.14 (0.08–0.24)**	**0.43**	**0.25**	**0.73**	**0.22 (0.12–0.37)**	**0.46**	**0.26**	**0.80**
C	**0.24 (0.14–0.40)**	**0.46**	**0.27**	**0.77**	**0.38 (0.22–0.66)**	**0.53**	**0.31**	**0.91**
B	**0.41 (0.24–0.69)**	**0.58**	**0.34**	**0.97**	0.78 (0.45–1.35)	0.76	0.45	1.29
A	1	1			1	1		
**Depressive symptoms perception**								
No	1.03 (0.92–1.15)	0.91	0.80	1.02	**1.27 (1.15–1.41)**	0.95	0.84	1.08
Yes	1	1			1	1		
**Behavioral lability perception**								
No	**1.19 (1.04–1.37)**	1.05	0.90	1.21	**1.35 (1.22–1.49)**	1.04	0.919	1.18
Yes	1	1			1	1		
**Disabilities**								
No	**1.64 (1.27–2.13)**	**1.31**	**1.03**	**1.66**	**1.547 (1.34–1.84)**	1.18	0.99	1.40
Yes	1	1			1	1		
**General health perception**								
Good	**3.92 (2.31–6.65)**	**2.32**	**1.38**	**3.90**	**3.67 (2.93–4.60)**	**2.04**	**1.60**	**2.61**
Regular	**1.98 (1.15–3.42)**	**1.84**	**1.08**	**3.15**	**2.03 (1.60–2.56)**	**1.55**	**1.21**	**1.99**
Poor	1	1			1	1		
**Restful sleep**								
Yes	1.09 (0.97–1.22)	1.09	0.973	1.23	**1.35 (1.21–1.50)**	**1.23**	**1.08**	**1.40**
No	1	1			1	1		
**Number of NCDs**								
1 NCD					**1.36 (1.14–1.62)**	0.86	0.71	1.05
2 NCDs					**1.30 (1.07–1.58)**	0.94	0.76	1.15
3 NCDs					1.06 (0.85–1.34)	0.84	0.66	1.07
4 or more NCDs					1	1		
**Housing**								
Urban	**2.22 (1.92–2.56)**	**1.44**	**1.23**	**1.68**	**2.09 (1.70–2.56)**	**1.38**	**1.08**	**1.75**
Rural	1	1			1	1		

Significant values are in bold.

NCDs, Noncommunicable diseases; CI-95%, 95% confidence interval; PR, Prevalence ratio; BMI, Body Mass Index.

It is noteworthy that illiterate or literate individuals are less active than those with higher education levels. It was also found that those who live in the urban area are more active (PR_nonNCD_ = 1.44; PR_NCD_ = 1.38) as well as those who have good (PR_nonNCD_ = 2.32; PR_NCD_ = 1.60) and regular (PR_nonNCD_ = 1.84; RP_NCD_ = 1.21) health perception. In contrast, smoking (PR_nonNCD_ = 0.64; PR_NCD_ = 0.69), drinking (PR_nonNCD_ = 0.77; PR_NCD_ = 0.77), live with spouse (PR_nonNCD_ = 0.67; PR_NCD_ = 0.85) and having paid work (PR_nonNCD_ = 0.87; PR_NCD_ = 0.89) are less associated with active style in the investigated groups (Table 2).

In the non-NCD group, a discrepancy between genders is observed, with male being 42% more active than females (PR = 1.42). This is also seen among those who have health insurance (PR = 1.26) and those who are not physically or mentally disabled (PR = 1.31). In contrast, in the NCD group, it is possible to identify a distinction between elderly and non-elderly people (PR_non-elderly_ = 1.22), which are more active as well as non-white people (PRwhite = 0.85) and those who have restful sleep (PR = 1.23) (Table 2).

In addition, the variables of informal social support and social class are related to the active lifestyle differently in the analyzed groups. The active lifestyle is associated with people with NCD only when they have a broad social support network, that is, above 6 to 10 people (PR_NCD_ = 1.46). In contrast, physically active people and non-NCD are more frequent when there are social support networks below 6 to 10 people (PR_non-NCD_ = 1.26) (Table 2). As for social class, all strata below class A are less associated with active lifestyle. The non-NCD group showed an increasing gradient of association with the active lifestyle between the strata DE up to A. On the other hand, in the NCD group, only strata DE (PR = 0.43) and C (PR = 0.56) differs from stratum A, which is similar to stratum B (Table 2).

Having perceptions of depressive symptoms (PR = 0.91/PR = 0.95) and emotional lability (PR = 1.05/PR = 1.04) as well as the number of NCDs in the group with these conditions were not associated with active style nor were they able to discriminate against them (Table 2).

## Discussion

It is necessary to understand the biopsychosocial inequalities that are related to the active lifestyle and that may potentiate the development of chronic diseases or worsen them. It is possible to state that in Brazil, among people with and without chronic health conditions, the proportion of physically active people is similar. However, there are biopsychosocial factors associated with the active lifestyle that sometimes are shared by both groups and some other factors are exclusively shown in only one of them.

One of these factors that revealed an association with active behavior in both groups was informal social support, however, with a different influence potential in both groups. Concomitantly, the more social support received, the greater the engagement of these people in physical practices, which can influence the prevention/management of NCDs. In this perspective, Wang et al. 2020^22^ evaluated the effect of social support on the level of physical activity, finding that the greater the support from family, friends and partners, the better the level of physical activity and the greater the self-efficacy. The specific social support for physical activity is an important factor that helps mainly the elderly to be physically active^23^, which is the case of the NCD group in this study, which comprises a higher proportion of elderly people.

Other biopsychosocial conditions that are related to active behavior in both groups are smoking and alcohol consumption. These habitsare less associated with active lifestyle in our study, which also contributes to the decline in quality of life and health, with a higher prevalence of chronic diseases, mainly in men^24^. People who smoke are more likely to present respiratory symptoms and lower levels of physical activity than people who do not smoke^24,25^. Physical exercise is a strong ally in the process of smoking cessation, because the practice of physical exercises provides positive effects in reducing withdrawal symptoms and impulsivity, thus suggesting a double effect direction and the need to encourage adherence to an active lifestyle^26^.

Similar to this situation, people who live in the urban area show higher prevalence in the practice of physical activity, when compared to the rural area, regardless of whether or not they have NCDs. Rural people inevitably have structural constraints, such as the absence of specific environments for the practice of physical activity, higher interaction with other determinants such as tobacco and alcohol abuse, the perception that human work is enough and diet, that is better than urban areas^27,28^, which can interfere in the understanding of the importance of the practice of planned physical activity as a protective health measure. This context reinforces the idea that the intensity of work can influence the practice of recreational physical activity^29^.

The individual's perception of feeling healthy or sick is not only due to physical sensations, but also to the social and psychological consequences of the disease^30^, and it is also a common condition for both groups with and without NCDs. In this study, it was observed that a perception of health as good or regular is associated with a more active lifestyle. It is a positive feedback, in which the more active a person is, the better the self-perception of one’s own health and vice versa^31,32^. This is an indicator of vitality and it must be measured individually and collectively in order to estimate how healthy the person and the population are^33^. Even with NCDs, individuals with a good perception of health are able to be more active, which may minimize their activity limitations and generate relevant social and economic impact^34^. The same occurs for those without NCDs, in this case the main idea is the prevention of these conditions.

Unlike the biopsychosocial characteristics discussed so far, the participant's sex also demonstrated influence, but only in the group without NCDs, where the sedentary pattern is more common in female individuals, compared to males. Considering the Brazilian adult population, 44.8% do not reach a satisfying level of physical activity, this percentage is higher among women than men^30^. Women experience more barriers to the practice of physical activity like race, age, income^34^ and motivation^35^ distinct from men, which is reduced according to the increase in the education level^33–36^. This was also one of our findings, when we observed that those with higher education or graduate degrees are more active.

This inequality does not seem to be related to biological but to social issues, as there is no evidence that the anatomophysiological composition of female individuals prevents them from having physically active behavior^34,35^. Furthermore, women who perceive and receive little social support related to the practice of physical exercise have a negative impact on quality of life, emphasizing that social support should be perceived as a determinant of people's health, by health services, by the community, through the several social actors, including family members, friends, neighbors, religious groups and health professionals^37,38^, which supports the statement that it is a gender issue and not biological conditioning.

Our findings indicate that age is only relevant for regular physical activity in individuals with NCDs, and the elderly in this group are less active than adults. A practice of weekly physical activity of moderate intensity tends to decrease with age and the presence of morbidities. The negative association between being elderly and active lifestyle in individuals with NCDs reported in this study is very worrying, considering that there is a great loss of physiological functions such as strength, balance and cognition that leads to the worsening of the progression stages of NCDs^38^, reinforced by the social stigmatization that elderly people suffer from the false assumptions of their potential fragility for physical exercise, the need for permanent rest and that their functional independence is minimal^39^.

On the other hand, education levels and income levels seem to relate to active behavior in both groups and minimize the effect of age and other social determinants. In an extensive systematic review, most qualified articles also found a positive relationship between income, education and levels of planned physical activity, such as sports and leisure. In this way, hypothetically, it is possible to suggest that the encouragement and performance of planned physical practices such as leisure and well-being actions have a higher frequency among those with higher education because these people better understand the importance of this practice for physical and mental health and social. Complementarily, a higher income could provide easier access to goods and services for the planning and execution of practical activities of sport and leisure, as well as more time for inclusion of these programs in the routines of wealthier people^40^.

As well as age, there is a negative association between white people and active lifestyle in the NCD group. This is a paradoxical condition, like gender perspective, because white people are more associated with better health status, but it might be explained by the interaction with the age, in which white elderly people have a higher life expectancy and, therefore, a greater chance of having NCDs^40^.

Having private health insurance is more associated with active behavior in the non-NCD group, and people with private health insurance generally belong to a high socioeconomic level and generally have a higher level of education. However, high social status and education are commonly associated with an active lifestyle in both groups in this study. Probably, the highest proportion of elderly people in the NCDs group prevent them from having access to insurance due to the higher fees charged for this age group, which does not make the association in this group significant. Another plausible scenario is that a quick and wide access to the diversified option of health professionals in specialized health services made possible by private insurance may facilitate adherence to the physically active lifestyle, which results in higher levels of physical activity only in the group without NCDs^34,41,42^.

On the other hand, people with paid work appear to be less active in both groups. This fact can be justified by having a more active life in the work aspect, which already promotes some type of physical effort, discarding the regular practice of physical activity prescribed as a preventive measure. Thus, it is essential to understand the barriers to the practice of physical activity in workers that could contribute to the elaboration of strategies for increasing the active lifestyle and improving quality of life in health promotion programs^44^ addressing specific skills of movement, control and self-regulation that come from the health and physical domains^43^.

The negative association between people with physical or intellectual disabilities and active lifestyle was found in the non-NCD group. People with disabilities who have more chances for development NCD due physicial inactivity. Another result shows significant increase in sedentary lifestyle, overweight and obesity in this population with disabilities, further increasing the chances of developing NCDs^46^.

In the NCD group, physical or intellectual disabilities can have their effects mixed with chronic morbidities, not being an inequality factor, mainly because the number of NCDs was not associated with active style. However, several barriers stand out, such as problems with the sidewalks, lack of appropriate facilities/spaces, lack of support policies for public entities, need for a guide, lack of provision of activities by specialized institutions and lack of security conditions of the facilities to avoid accidents^46^. More studies are needed in this population regarding the levels of physical activity.

Studies show that higher levels of physical activity are related to greater efficiency and quality of sleep^44^. Restful sleep was positively associated with an active lifestyle only in the NCD group. Sleep plays a fundamental role in the self-perceived health status^45^ and physiological recovery and people with NCDs suffer extensively with symptoms of insomnia and excessive daytime sleepiness mediated by medications, pain and physiological changes in the sleep–wake cycle^46,47^.

Reports by Silva and Boing^48^ and Mielke et al.^49^ use the same database and have similar purposes to our study. However, these studies are limited to data analysis and make mistakes that compromise the inference. The study by Silva and Boing only considered individuals diagnosed with hypertension, diabetes and/or hypercholesterolemia, whereas our study analyzes the entire spectrum of NCDs with more than 20 diseases. In addition, that study makes an important error in estimating the association between outcome and factors based on the odds ratio (OR) by logistic regression. When the outcome has high prevalence (> 10%), OR cannot be used as a proxy for the prevalence ratio (PR) because point estimates are oversized and interval estimates are extended, producing inaccurate results. The best alternative would be a robust Poisson or Cox regression. As for the study by Mielke et al.^49^, this is a descriptive study, only presenting the magnitude of physical activity practice and sedentary habits in the sample of adults, without raising any inference about the factors implicating these behaviors.

Despite inferences of great impact shown in this study, there are some limitations that must be highlighted. The first one is due to the self-reported characteristic of NCDs whose accuracy varies according to the type of health condition and the participant's literacy^50^. The second limitation is related to the cross-sectional design, that makes it difficult to establish causality for temporally uncertain characteristics. The selection bias was possible, as only those available in their households at the moment of the interviews were selected. Finally, the limitation of not identifying the intensity of physical activity between the groups, not knowing whether there is a distinction between the moderate or vigorous level of exercise, even though knowing that both intensities generate health benefits.

## Conclusion

Biological and psychosocial factors are unevenly associated with the practice of physical activity between groups of people with and without NCD. Thus, proposals for public health policies actions for the promotion, prevention or care of health, must observe individual and collective specificities before developing guidelines and actions to encourage and carry out physical practices among people.

Treating these subpopulations equally may generate inequities for the active style and inefficacy of results, especially in individuals with NCDs, who tend to be more affected in the presence of social injustices than those without NCDs. Qualified equitable approaches focusing on smoking and drinking cessation, on individuals from rural areas, low social strata and low education levels are essential for health promotion.

## Supplementary Information

Supplementary Information.
