# Supplementary material for: Biopsychosocial inequality, active lifestyle and chronic health conditions: a cross-sectional National Health Survey 2013 in Brazil
Source: Sci Rep. 2021 Dec 14;11:24010. doi: 10.1038/s41598-021-03549-5 (PMC8671513; doi:10.1038/s41598-021-03549-5)
Supplement: Supplementary file 1 — Supplementary Information. [file 41598_2021_3549_MOESM1_ESM.docx]

SUPPLEMENTARY TABLE 1 – List of selected chronic non-transmissible disease of the 2013 national health survey in Brazil.

| **Cardiovascular and metabolic** | **Respiratory** | **Musculoskeletal** | **Mental Health** | **Cancer** | **Disabilities** |
| --- | --- | --- | --- | --- | --- |
| Arterial hypertension | Asthma (or asthmatic bronchitis) | Arthritis or rheumatism | Depression | Lung | Intellectual disability   - Down syndrome - Autism - Cerebral palsy |
| Diabetes | Lung disease   - Pulmonary emphysema - Chronic bronchitis - COPD (Chronic obstructive pulmonary disease) | Chronic back disease   - Neck or back pain - Low back pain - Sciatic pain - Disc or vertebra problem | Schizophrenia | Intestine | Physical disability   - Permanent paralysis on one side of the body - Permanent paralysis of the legs and arms - Permanent paralysis of the legs - Permanent paralysis of one of the legs - Amputation or absence of leg - Amputation or absence of an arm - Amputation or absence of hand - Amputation or absence of foot - Congenital or acquired deformity in one or more limbs - Motor disability due to polio or childhood paralysis - Ostomy (stool and/or urine adaptation) - Dwarfism |
| hypercholesterolemia |  | WMSD (work-related musculoskeletal disorder) | Bipolar disorder | Stomach | Hearing disability   - Deafness in both ears - Deafness in one ear and reduced hearing in the other - Deafness in one ear and normal hearing in the other - Reduced hearing from both ears - Reduced hearing in one ear |
| Infarction, angina, heart failure or other |  |  | Psychosis | Cervix | Visual impairment   - Blindness of both eyes - Blindness in one eye and reduced vision in the other - Blindness in one eye and normal vision in the other - Low vision in both eyes - Low vision in one eye |
| Stroke |  |  | OCD (Obsessive-compulsive disorder) | Prostate |  |
| Chronic kidney failure |  |  |  | Skin |  |

**Caption:** table containing the non-communicable chronic diseases considered in this study. Clinical conditions were subclassified into conditions Cardiovascular and metabolic, Respiratory, Musculoskeletal, Mental Health, Cancer and Disabilities.
